# Adolescents With Congenital Heart Disease: What Do They Know About Reproductive Health and Risks?

**DOI:** 10.1097/JCN.0000000000000838

**Published:** 2021-07-06

**Authors:** Åsa Burström, Mariela Acuña Mora, Carina Sparud-Lundin, Philip Moons, Ewa-Lena Bratt

**Affiliations:** **Åsa Burström, PhD, RN** Lecturer, Department of Pediatric Cardiology, Astrid Lindgren Children's Hospital, and Department of Neurobiology, Care Science, and Society, Karolinska Institutet, Stockholm, Sweden.; **Mariela Acuña Mora, PhD, RN** Lecturer, Institute of Health and Care Science, University of Gothenburg, and Faculty of Caring Science, Work Life and Social Welfare, University of Borås, Sweden.; **Carina Sparud-Lundin, RN** Professor, Institute of Health and Care Science, University of Gothenburg, Sweden.; **Philip Moons, RN** Professor, Institute of Health and Care Science, University of Gothenburg, and KU Leuven, Department of Public Health and Primary Care, Leuven, Belgium.; **Ewa-Lena Bratt, RN** Associate Professor, Institute of Health and Care Science, University of Gothenburg, and The Queen Silvia Children's Hospital, Gothenburg, Sweden.

**Keywords:** adolescents, congenital heart disease, counseling, reproductive health

## Abstract

Studies show that adolescents with congenital heart disease (CHD) lack knowledge about reproductive health in relation to their CHD.

**Objective:**

The aims were to investigate (i) the proportion of adolescents with CHD receiving information about reproductive health, (ii) the level of reproductive health knowledge in adolescents with CHD, and (iii) potential correlates for receiving information about reproductive health.

**Methods:**

A total of 202 adolescents aged 14 to 18 years (mean age 15.7 ± 1.1 years) with CHD completed the Knowledge Scale for Adults with Congenitally Malformed Hearts and were asked if they had received information about contraception and pregnancies or if this would be of interest.

**Results:**

Few adolescents could recall receiving information about contraceptives (5%) and pregnancies (15%). Furthermore, only 24% adolescents wanted information about contraceptives, and 42% of the female adolescents wanted information about pregnancies. There was a higher probability of male adolescents wanting information about contraceptives. Knowledge about reproductive health varied regarding knowledge about why they had been born with CHD (68%), knowledge about the hereditary nature of the CHD (48%), and if sexual activity could worsen their CHD (70%). However, few (11%) had knowledge about the elevated risk of having a child with CHD. Age was associated with a higher probability of having knowledge about the risks of having a child with CHD.

**Conclusions:**

The low number of adolescents receiving information about contraceptives and pregnancies may have implications for future health and family planning. Future research is needed to identify and evaluate successful communication strategies that help to identify adolescents' preferences on how to approach this sensitive topic.

Medical and surgical improvements have resulted in improved long-term survival among children with congenital heart disease (CHD), and today, almost 90% reach adulthood.^1^ Accordingly, an increasing number of young persons with CHD are reaching adolescence and reproductive age. Because many of these adolescents will become sexually active, there is an increasing need to provide better reproductive health knowledge to prevent poor outcomes regarding contraception or pregnancy.

Adolescence is a developmental life phase characterized by several physical, cognitive, social, and emotional changes.^2^ During adolescence, identity is developed by exploring and experiencing different peer/love relationships and different educational and vocational opportunities for a longer period before committing to long-term choices.^3^ Moreover, experimenting behaviors are a natural part of adolescence, but for young persons with CHD, risky behaviors may have long-term effects on their future health and life.^4,5^ During this developmental transition into adulthood, adolescents with CHD need to learn how to manage their health, including reproductive health.^6^

Previous studies have revealed that adolescents with CHD lack knowledge about reproductive health in relation to their CHD.^7,8^ This knowledge gap is still prevalent in adults with CHD.^9,10^ As a consequence, this may lead to unnecessary fear and anxiety regarding pregnancy, childbirth, and future offspring,^10^ as well as unnecessary exposure to potential risks associated with choice of contraceptive.^11–13^ A lack of knowledge about effective contraceptives might also lead to unwanted pregnancies.^14^ Given the heterogeneity of CHD, reproductive health recommendations will vary greatly. Whereas some patients will require specific contraceptives that decrease the risk of adverse events such as thrombosis, others will require frequent follow-up during pregnancy. In particular cases, the risk of developing complications will be higher.^15,16^

Adolescents with CHD describe concerns about reproductive issues of relevance for their CHD^6^ and a desire to discuss the topic with their healthcare provider.^10,17^ Young people with CHD need counseling about reproductive health significant to their CHD to facilitate the establishment of safe intimate relationships and family planning.^16,18^

The healthcare provider can provide tailored and timely information and adapt it to the adolescents' level of maturity by assessing their concerns and knowledge about reproductive health.^10^ Such information can prevent adverse events associated with reproductive health issues in adolescents with CHD. However, the first step is to investigate what reproductive health knowledge adolescents with CHD actually have and to explore potential associated factors.

## Objectives

The objectives of this study were to investigate the following:

(i) the proportion of adolescents with CHD receiving information about reproductive health
(ii) the level of reproductive health knowledge in adolescents with CHD
(iii) potential correlates for receiving information about reproductive health

## Methods

Data were from a cross-sectional, multicenter study that is part of the Stepstones project (Swedish Transition Effects Project Supporting Teenagers With Chronic Medical Conditions).^19^

### Settings and Participants

The participants were recruited from 4 university hospitals in Sweden. Potential participants were adolescents aged 14 to 18 years with a CHD and under current follow-up. Exclusion criteria were adolescents with syndromes affecting cognitive abilities, heart transplantation, and acquired heart diseases; who were illiterate; or who were non-Swedish speaking. The Swedish registry of CHD^20^ was used to identify all eligible adolescents fulfilling the inclusion criteria. A total of 593 adolescents were identified as fulfilling the inclusion criteria.

### Data Collection

Data were collected through questionnaires from January to August 2016. Study information, informed consent form, and the questionnaires, along with a preaddressed envelope, were sent to eligible participants and their parents. Study consent was obtained from both adolescents and parents, even though in Sweden, young persons from the age of 16 years can be approached without parental consent. To improve the response rate, reminders were sent by post after 3 and 5 weeks and if no response was received, a final reminder by telephone was given after 7 weeks.^21^ Ethical approval was granted by the Ethical Board, Gothenburg (Diary no. 953-13), and the study was performed according to the Declaration of Helsinki.^22^

### Variables and Measurement

Sociodemographic data, along with the questionnaires, were collected from the participants. Clinical characteristics were retrieved from the medical records. The complexity of the CHD was classified into mild, moderate, and complex lesion according to Bethesda Conference Task force 1 classification. The classification indicates the complexity of the CHD and the need for care.^23^

The Knowledge Scale for Adults With Congenitally Malformed Hearts (KnoCoMH) was used to collect data on knowledge about reproductive issues. This scale's psychometric properties have been shown to be acceptable for an adult population.^24^ The original KnoCoMH was inspired by and developed from the Leuven Knowledge Questionnaire for Congenital Heart Disease, which has been tested for psychometric properties and shown to be satisfactory for a population of adolescents.^25,26^ The research group discussed the content of the instrument (KnoCoMH) before using it in this study. To improve face validity among adolescents, cognitive interviewing was performed such that the adolescents were asked to read through the questionnaire. The adolescents were then individually interviewed and asked to comment the questions. The questionnaire was easy to understand but there were some adjustments needed to make the scale more understandable. These adjustments were related to expressions that seemed to be too adult oriented. In addition, a few items related to endocarditis were removed because the medical recommendations have changed.^27^ The modified scale contains 19 items that comprise 4 domains concerning general knowledge, medical treatment, endocarditis, and pregnancy. In the present study, only 4 items from the modified KnoCoMH were used: knowledge about why born with a CHD, knowledge about heredity, knowledge about elevated risk of having a baby with CHD, and knowledge about risks of worsening the CHD due to sexual activity. These items had multiple-choice possibilities. The response alternatives were dichotomized as correct or incorrect (“do not know” was categorized as incorrect).^24^

Four items were added to address whether the adolescents had received information about contraceptives (yes/no) and whether the female participants had received information about pregnancy from their healthcare provider in conjunction with their medical check-up (yes/no). If the answer was no to either of these 2 questions, they received a follow-up question regarding whether they wanted information (yes/no). Items regarding information about pregnancy were addressed only to female participants. In total, female participants could potentially answer 8 items, whereas male participants could respond to 6 items (Table 1).

**TABLE 1:** Items Regarding Knowledge and Information

Items regarding receiving information
Have you received information about contraceptives from your doctor or nurse while visiting the heart unit? Yes/No
If not, is this something you'd like?
Have you received information from your doctor or nurse considering future pregnancies in relation to your CHD?^a^ Yes/No
If not, is this something you'd like?^a^
Items from the KnoCoMH questionnaire
Do you know why you were born with a CHD?
a) Something happened during the development of my heart when I was still in my mother's womb (during the fetal period), which led to a heart defect
b) Something happened to my heart when I was newborn
c) Do not know
d) Other…
Is your CHD hereditary?
a) No, my congenital heart defect is not hereditary
b) Yes, my congenital heart defect is hereditary
c) Do not know
Can sexual activity worsen your CHD condition?
a) No
b) Yes
c) Do not know
How great is the risk that your child(ren) will have a CHD?
a) No increased risk
b) Insignificantly increased risk
c) Moderately increased risk
d) Significantly increased risk

Abbreviations: CHD, congenital heart disease; KnoCoMH, Knowledge Scale for Adults with Congenitally Malformed Hearts.

^a^Question addressed only to female participants.

### Statistical Analysis

Data were analyzed using IBM SPSS statistics for Windows version 27 (IBM Corp, Armonk, New York). Descriptive statistics were expressed in absolute numbers and percentages for nominal variables or in means and standard deviations for continuous variables.

We used multivariable logistic regression analysis (enter method) to estimate which factors were associated with receiving information or with knowledge about reproductive health. By using the enter method, all covariates are included at the same time when undertaking the regression analyses. We used 3 explanatory variables (age, sex, and CHD complexity) in the regression analysis. For the analyses concerning receiving information about pregnancy, only data from the female participants were used.

Odds ratios (ORs) with 95% confidence intervals were computed in the model. A *P* value <.05 was considered indicative of statistical significance and all tests were 2 sided.

## Results

A total of 202 (91 female) adolescents (34% response rate) with a mean (SD) age of 15.7 (1.1) years (range, 14–18 years) participated. Thirty-two percent of the adolescents had a mild CHD, 42% had moderate CHD, and 27% had severe CHD (Table 2). See Table 2 for information on demographics and medication.

**TABLE 2:** Demographics of the Participating Adolescents

	n (%)
Age, mean (SD), y	15.7 (1.1)
Gender (n = 202)	
Female	91 (45)
CHD complexity (n = 202)	
Mild	63 (32)
Moderate	84 (42)
Severe	55 (27)
Current education level (n = 195)	
Elementary	112 (57)
High school	81 (42)
Other	2 (1)
Living conditions (n = 201)	
Living with both parents	155 (77.)
Living alternately with mother and father	18 (9)
Mother	20 (10)
Father	4 (2)
Other	4 (2)
Taking medication for your heart condition? (n = 201)	44 (22)
Born with other congenital condition? (n = 196)	34 (17)
Needing treatment for any other condition? (n = 195)	29 (15)

Abbreviation: CHD, congenital heart disease.

### Information About Reproductive Health

Few adolescents could recall receiving information from their healthcare provider about contraceptives or pregnancies and the proportion varied according to age, complexity, and sex. Only female participants (n = 7) and adolescents older than 16 years had received information about contraceptives. Regarding adolescents who denied receiving information, only 31 reported an interest in receiving it (Table 3). There was a higher probability of male participants wanting information about contraceptives (OR, 4.8; 95% CI, 1.6–14.2; *P* = .004), and CHD complexity and age were not significant (Table 4).

**TABLE 3:** Information About Contraceptives and Pregnancy, and Knowledge About Heredity and Sexual Activity

	Have You Received Information About Contraceptives?	If Not, Is This Something You Want?	Have You Received Information About Future Pregnancies?^a^	If Not, Is This Something You Want?^a^	Knowledge About Why Born With a CHD	Knowledge About Heredity	Can Sexual Activity Worsen Your CHD?	Elevated Risk of Having a Baby With CHD?
Answer	Yes	Yes	Yes	Yes	Correct	Correct	Correct	Correct
CHD complexity, n (%)								
Mild	1 (2)	9 (20)	0	10 (31)	30 (48)	20 (32)	45 (75)	2 (3)
Moderate	4 (7)	18 (35)	7 (17)	18 (54)	62 (76)	40 (49)	56 (68)	10 (12)
Severe	2 (6)	4 (12)	6 (43)	3 (33)	42 (79)	36 (67)	35 (66)	9 (17)
Total, n (%)^b^	7 (5)	31 (24)	13 (15)	31 (42)	134 (68)	96 (48)	136 (70)	21 (11)
Age								
14 y	0	5 (19)	1 (83)	5 (46)	21 (57)	18 (49)	22 (65)	1 (3)
15 y	0	7 (23)	2 (11)	6 (38)	33 (70)	23 (48)	30 (64)	2 (4)
16 y	1 (3)	9 (30)	3 (12)	8 (38)	30 (64)	20 (43)	31 (66)	6 (13)
17 y	6 (13)	10 (22)	7 (21)	12 (46)	50 (77)	35 (53)	53 (79)	12 (18)
Total n (%)^b^	7 (5)	31 (24)	13 (15)	31 (42)	134 (68)	96 (48)	136 (70)	21 (11)
Sex								
Female	7 (8)	26 (33)			60 (70)	43 (49)	58 (67)	14 (16)
Male	0	5 (10)			74 (67)	53 (48)	78 (72)	7 (7)
Total n (%)^b^	7 (5)	31 (24)			134 (68)	96 (48)	136 (70)	21 (11)
Number of responders	140	131	87^a^	74^a^	196	198	195	195

Abbreviation: CHD, congenital heart disease.

^a^Items regarding receiving information about pregnancy were answered only by female participants.

^b^The results under each subsection signify the number and percentage of adolescents who answered yes or correctly to the questions.

**TABLE 4:** Factors Associated With Receiving Information on Contraceptives and Pregnancy and on Knowledge About Reproductive Health: Multivariate Logistic Regressions Analysis

	Have You Received Information About Contraceptives?	If Not, Is This Something You Want?	Knowledge About Why Born With a CHD	Knowledge About Heredity	Can Sexual Activity Worsen Your CHD?	Elevated Risk of Having a baby With CHD?	Have You Received Information About Pregnancy?^a^	If not, Is This Something You Want?^a^
CHD complexity								
Severe vs mild	0.82 (0.11–6.1) *P* = .85	3.5 (0.09–13.2) *P* = .07	0.56 (0.24–1.3) *P* = .56	0.6 (0.28.1.3) *P* = .19	0.86 (0.34–2.2) *P* = .75	1.5 (0.38–5.9) *P* = .57	0.34 (0.7–1.7) *P* = .19	1.5 (0.35.6.1) *P* = .6
Severe vs moderate	1.3 (0.18–9.4) *P* = .79	0.84 (0.03—2.4) *P* = .75	(0.51–2.5) *P* = .76	0.81 (0.40–1.6) *P* = .56	0.45 (0.2–1.0) *P* = .057	2.4 (0.72–8.0) *P* = .15	4.5 (0.99–20.3) *P* = .052	0.05 (0.14–2.0) *P* = .75
Age	0.16 (0.02–1) *P* = .06	1.2 (0.75–1.8) *P* = .47	1.2 (0.92–1.6) *P* = .16	(0.77–1.3) *P* = 1	1.3 (0.94–1.7) *P* = .12	2.2 (1.2–3.9) *P* = .007	0.81 (0.42–1.6 *P* = .5	0.92 (0.57–1.5) *P* = .34
Sex								
Female vs male participants	103633797 (0.0-.) *P* = .99	4.8 (1.6–14.2) *P* = .004	0.95 (0.51–1.8) *P* = .89	0.97 (0.55–1.7) *P* = .93	1.4 (0.74–2.7) *P* = .3	.44 (0.17–1.2) *P* = .1		
Nagelkerke *R*^2^	0.33	0.18	0.05	0.01	0.07	0.14	0.11	0.02

Data are EXP(*B*) (95% CI).

Abbreviations: CHD, congenital heart disease; CI, confidence interval.

^a^Answered only by female participants.

^b^Only 7 respondents (only girls)/140 answered the question.

Regarding information about pregnancy given to the female participants, only 15% could recall receiving such information, and there was a variety of ages and CHD complexity in this cohort. Of the female participants who denied receiving information, almost half (42%) wanted to have information from the healthcare provider (see Table 3).

### Knowledge About Reproductive Health

The results showed that many adolescents (68%) had knowledge about why they had been born with a CHD. Moreover, about half the adolescents (48%) had knowledge about the hereditary nature of the CHD and 70% could correctly answer if sexual activity could worsen their CHD condition. However, only 11% had knowledge about the elevated risk of having a child with CHD (see Table 3). Moreover, there was a higher probability of older adolescents having knowledge about the elevated risk of having a child with CHD (OR, 2.2; 95% CI, 1.2–3.9; *P* = .007), but sex and CHD complexity were not significant (see Table 4).

## Discussion

The results of this study show that few adolescents could recall talking about contraception or pregnancy with their healthcare provider, and this has also been reported in studies on adults with CHD.^9,28,29^ However, these findings are contrary to international recommendations, which emphasize preventive health counseling that includes reproductive health for adolescents with CHD,^30^ particularly as intimate relationships are a common aspect of adolescent life.^31^ This underlines the importance of starting to discuss contraception and preconception with these adolescents from an early age and in a developmentally appropriate way to prevent unplanned pregnancies and encourage safe contraceptive methods.^30^

An interesting finding was that only a quarter of the adolescents in the present study wanted information about contraceptives. This finding contrasts with a previous interview study^17^ where the need for information about contraceptives and pregnancies was emphasized. One possible explanation could be the presence of youth clinics in the Swedish healthcare system, available to all young people. These youth clinics are easily accessible, and the adolescents have the opportunity to get counseling on reproductive issues or make appointments with midwives or psychosocial workers. Another possible explanation for neglecting to ask for information might be concerns about discussing sensitive topics with healthcare provider.^32^ Only 70% of the adolescents in the current study answered the question if they had received information about contraceptives. This might reflect and underline that it is a topic difficult to talk about not only for young people but also for the healthcare provider. According to a systematic review by Kim and White, adolescents can be embarrassed when discussing their own sexuality, and conversely, healthcare provider can feel awkward when talking about these issues with them. Kim and White suggest developing resources and training for healthcare provider to improve communication.^33^ However, concerns regarding confidentiality may be another important factor. Confidentiality is a major factor contributing to adolescents feeling safe when discussing reproductive health with their healthcare provider. The American Academy of Pediatrics encourages tailored counseling that is individualized according to behaviors, values, and goals and engages the adolescent in their care in an emphatic and nonjudgmental way.^31^ If confidentiality is not assured, there is no willingness to disclose sensitive topics. For adolescents, being assured of privacy and confidentiality when sharing sensitive health information is very important.^34^

Few female adolescents could recall receiving information about pregnancies. Cardiologists and nurses within the pediatric setting can easily overlook this topic, one possible reason being that the healthcare provider perceives the patients within the pediatric setting to be too young. But previous studies have indicated that young women (aged 15–19 years) with CHD are interested in information about pregnancy and are concerned about fertility and future offspring.^10,35^ Notably, however, the results of our study show that less than half of the female participants were interested in receiving information. Healthcare providers may experience barriers such as time constraints, practice, and knowledge that hinder reproductive counseling.^9^ However, although the adolescent may not be planning a pregnancy during their teenage years, upon getting pregnant, they hopefully will recall a need for precounseling.^30^ In conversations with younger adolescents, pregnancy topics should be focused on special considerations and risks (or no risks) regarding pregnancy in relation to the adolescent's specific type of CHD and actual treatment. One could argue that if the adolescents are aware that a pregnancy could be a strain for the heart, they could be encouraged to have a preconception talk with their cardiologist healthcare provider before planning a pregnancy. During adolescence, such topics should be introduced in an age and developmentally appropriate way.^17^

The relatively low number of adolescents who wanted to talk about contraceptives could also be indicative of a lack of awareness regarding the possible risks associated with different types of contraceptives. The Committee on Adolescence and American Academy of Pediatrics underscores the importance of talking about sexual health and contraception with the adolescents in their consultations. They argue that adolescents often find healthcare providers to be trusted sources who can support them in making healthy and informed decisions.^31^ It is important to keep in mind that, although an adolescent may have had such discussions with other healthcare providers, they will need to get adequate information about these issues from a medical perspective because of their CHD.^36,37^ Living with a CHD while being unaware of the contraindications or side effects can put an adolescent at risk. A study in a pediatric center in the United States found that only a third (28.6%) of adolescents with prescribed teratogenous medication had received counseling about contraceptives to protect against unplanned or unwanted pregnancies.^38^ Moreover, although one reason for using hormonal contraception is menstrual dysfunction, it has been reported that not all young females with CHD are aware of the contraindications of these drugs for their condition.^39^

Knowledge about why they were born with a CHD, about heredity, about elevated risk of having a baby with CHD, and about the risk of worsening their CHD due to sexual activity varied among the adolescents. The present study showed that the proportion of adolescents with mild CHD who knew about hereditary aspects was less than those with more complex CHD, although there was no significant association regarding CHD complexity, sex, and age. One possible explanation for the difference in proportion could be that the interval for the medical follow-up is dependent on the severity of the CHD.^40,41^ That means that adolescents with more complex CHD meet their healthcare provider more frequently than those with milder CHD. Females with milder CHD are considered to be at a minor risk of adverse events, which might be why discussing these is less of a priority during their medical appointments. However, this may lead to adolescents living with a misconception regarding reproductive health. To address this, information about reproductive health should be included when the adolescents attend their medical appointments, especially as these meetings are few and far between. If the healthcare provider overlooks the opportunity to discuss the topic, it is possible that adolescents will fail to learn about reproductive health in relation to their CHD.^18^

It is during this sensitive period in life and during the transfer from pediatric to adult care that adolescents need to prepare to take responsibility for their health and healthcare. This includes reproductive health, and it is important that healthcare provider support this matter. Specialist nurses are well equipped to have a coordinating role in supporting the adolescents in medical and health management, including reproductive health issues.^42,43^ In a nurse-led consultation, the adolescent has an opportunity/possibility in a person-centered way to learn about the CHD and day-to-day disease management.^44^ By using a psychosocial interview guide, such as HEADSS (Home, Education, Employment, Activities, Drugs, Sexuality, and Suicide), the nurse (and other healthcare providers) can ensure that important aspects of adolescent life and environment are covered, including sex and contraception. This interview guide is a practical tool that helps the healthcare provider to structure questions to maximize communication and reduce the stress that can arise when sensitive topics are to be addressed. The HEADSS is a support when creating a safe communication environment.^45^

### Limitations and Considerations

There are some limitations and considerations in this study that need to be discussed. First, the KnoCoMH questionnaire was developed and validated in adults with CHD and not in adolescents. However, the Leuven Knowledge Questionnaire for Congenital Heart Disease, from which the KnoCoMH was developed, has been validated and considered valid in an adolescent population. In addition, the KnoCoMH questionnaire was discussed within the research team and with a pediatric cardiologist, and its face validity was confirmed in a group of adolescents with CHD. Second, the adolescents' responses were verified and classified according to their main diagnosis in the medical record, and no consideration was given to comorbidities. Third, there was a low response rate, as only one-third of the eligible patients with CHD in the 4 centers participated, which might affect the generalizability of the study.

## Conclusions

The low number of adolescents receiving information about contraceptives and pregnancies may have implications for future health and family planning. Insufficient knowledge can lead to unfounded fear of family planning, but at the same time, it can lead to young women with complex CHD exposing themselves to avoidable risk. Few adolescents could recall having received information about contraceptives or pregnancies, which is contrary to international recommendations, and less than half of the adolescents claimed that they wanted information about contraceptives or pregnancies. Congenital heart disease complexity and age could not explain the likelihood of receiving information about reproductive health. Reproductive health knowledge varied among the participants. Reproductive knowledge could not be explained by CHD complexity, age, or sex, except for knowledge about elevated risk of having a baby with CHD. Future research is needed to identify and evaluate successful communication strategies that help identify adolescents' preferences on how to approach this sensitive topic. Furthermore, research is needed to evaluate if healthcare communication with adolescents can be improved by incorporate interview guides like HEADSS in nurse-led consultations.

**What’s New and Important**

- Few adolescents had received information about contraceptives or future pregnancies, and contrary to previous interview studies, less than half of the adolescents were interested in it.
- Age, sex, and CHD complexity could not explain the likelihood of adolescents receiving information about contraceptives.
- Age and CHD complexity could not explain the likelihood of females receiving information about future pregnancies.
